# Oxidative Stress in Multiple Myeloma: Pathogenic Mechanisms, Biomarkers, and Redox-Targeted Therapeutic Strategies

**DOI:** 10.3390/ijms27073001

**Published:** 2026-03-25

**Authors:** Rafał Bilski, Daria Kupczyk, Karolina Kaczorowska-Bilska, Halina Tkaczenko, Natalia Kurhaluk, Tomasz Kosmalski, Artur Słomka, Renata Studzińska

**Affiliations:** 1 Department of Medical Biology and Biochemistry, Collegium Medicum in Bydgoszcz, Nicolaus Copernicus University, M. Karłowicz Str. 24, 85-092 Bydgoszcz, Poland; 2 Clinic of Hematology, Ludwik Rydygier Collegium Medicum in Bydgoszcz, Nicolaus Copernicus University in Toruń, K. Ujejskiego Str. 75. 85-168 Bydgoszcz, Poland; 3 Institute of Biology, Pomeranian University in Słupsk, Arciszewski Str. 22b, 76-200 Slupsk, Poland; halina.tkaczenko@upsl.edu.pl (H.T.);; 4 Department of Organic Chemistry, Faculty of Pharmacy, Collegium Medicum in Bydgoszcz, Nicolaus Copernicus University in Toruń, Jurasza Str. 2. 85-089 Bydgoszcz, Poland; tkosm@cm.umk.pl (T.K.); rstud@cm.umk.pl (R.S.); 5 Department of Hematology and Oncology, National Medical Institute of the Ministry of Interior and Administration, 02-507 Warsaw, Poland; artur.slomka@pimmswia.gov.pl

**Keywords:** multiple myeloma, oxidative stress, reactive oxygen species, PRDX6, biomarkers, proteasome inhibitors, CAPE, gut microbiota

## Abstract

Multiple myeloma (MM) is an incurable plasma cell malignancy characterized by high metabolic activity, chronic endoplasmic reticulum stress, and persistent redox imbalance. Excessive immunoglobulin synthesis and adaptation to the hypoxic bone marrow microenvironment lead to sustained production of reactive oxygen species (ROS). Their excessive accumulation promotes genomic instability, disease progression, osteolytic bone disease, and resistance to therapy. Paradoxically, MM cells adapt to oxidative stress by activating antioxidant and metabolic defense mechanisms, including Nuclear factor erythroid 2-related factor 2 (NRF2)- and Heme Oxygenase 1 (HMOX1)-dependent pathways, metabolic reprogramming, and overexpression of ROS-scavenging enzymes such as peroxiredoxin 6 (PRDX6), allowing survival at the threshold of oxidative toxicity. Evidence indicates that biomarkers of oxidative stress—such as lipid and protein oxidation products, antioxidant enzyme activity, and the Oxidative Stress Score—correlate with disease stage, prognosis, and treatment response. Redox-modulating therapeutic strategies, including pharmacological ROS induction, inhibition of antioxidant defenses, and the use of natural pro-oxidant compounds, are emerging as promising adjuncts to standard MM therapies. Recent studies also highlight the gut microbiota as an indirect regulator of oxidative balance, immune modulation, and metabolic homeostasis in MM. This review summarizes current knowledge on oxidative stress in multiple myeloma, emphasizing its role in pathogenesis, drug resistance, biomarker development, and emerging therapeutic and supportive strategies.

## 1. Introduction

Multiple myeloma (MM) is a cancer derived from clonal plasma cells, which, under physiological conditions, are responsible for antibody production [1]. The process of malignant transformation leads to uncontrolled proliferation of these cells in the bone marrow, overproduction of monoclonal immunoglobulin (so-called M protein), and damage to critical organs. The clinical presentation of MM is varied, but the classic symptoms are summarized in the abbreviation CRAB: hypercalcemia (C—calcium), renal failure (R—renal), anemia (A—anemia), and bone lesions (B—bone lesions) [2]. The disease accounts for approximately 10% of all hematological malignancies and 2% of all cancers, with a peak incidence between the ages of 65 and 70 [3,4]. Significant therapeutic progress has been made in the last two decades. The introduction of proteasome inhibitors (bortezomib, carfilzomib, ixazomib), immunomodulatory drugs (thalidomide, lenalidomide, pomalidomide), monoclonal antibodies (daratumumab, isatuximab, elotuzumab), and the possibility of autologous hematopoietic cell transplantation have increased the median survival of patients from approximately 3–4 years in the early 21st century to over 8–10 years today. Despite these advances, MM remains an incurable disease, as almost all patients relapse sooner or later, and myeloma cells develop multidrug resistance [5,6,7,8]. In searching for the causes of this resistance, researchers have turned their attention to oxidative stress. Oxidative stress, defined as an imbalance between reactive oxygen species (ROS) production and antioxidant capacity, arises from both intracellular metabolic processes, including mitochondrial respiration and endoplasmic reticulum protein folding, as well as environmental factors. While physiological ROS levels play an essential signaling role, their excess induces DNA, lipid, and protein damage, contributing to genomic instability, malignant transformation, and altered responses to anticancer therapy [9].

Myeloma cells are particularly exposed to oxidative stress due to several overlapping mechanisms. Excessive immunoglobulin synthesis leads to chronic endoplasmic reticulum stress and sustained ROS generation during oxidative protein folding [10,11]. Concurrently, hypoxic conditions within the bone marrow microenvironment promote mitochondrial dysfunction, stabilization of hypoxia-inducible factor-1α (HIF-1α), metabolic reprogramming, and induction of antioxidant genes such as Heme Oxygenase 1 (HMOX1), enabling adaptation to a ROS-rich environment [12,13,14]. In addition, oncogenic transformation and epigenetic alterations further deregulate redox homeostasis and cellular metabolism, perpetuating oxidative pressure in malignant plasma cells [15]. This creates a biological paradox in which oxidative stress, potentially cytotoxic under physiological conditions, is converted by myeloma cells into a driver of survival, progression, and therapy resistance [16].

The importance of oxidative stress in MM extends beyond cancer cell biology. Its effects are reflected in the clinical picture and patient prognosis: from disease progression, through the development of organ damage, to mechanisms of treatment resistance [17]. Therefore, research on markers of oxidative stress (such as malondialdehyde, advanced oxidation protein products (AOPPs), and the recently proposed Oxidative Stress Score) and therapeutic strategies targeting redox balance are currently among the most promising directions in MM research. This review summarizes current knowledge on oxidative stress in multiple myeloma, focusing on redox imbalance as a driver of disease pathogenesis, drug resistance, biomarker development, and emerging therapeutic strategies.

## 2. Redox Homeostasis and Oxidative Stress in Cancer Cells

ROS comprise both free radicals, such as superoxide anion (O_2_•^−^) and hydroxyl radical (•OH), and non-radical molecules, including hydrogen peroxide (H_2_O_2_). Under physiological conditions, ROS generation is tightly regulated, and moderate ROS levels play an essential role in intracellular signaling, regulating processes such as cell proliferation, differentiation, apoptosis, and immune responses. However, excessive accumulation of ROS results in oxidative damage to macromolecules—including DNA, proteins, and lipids—leading to genomic instability and activation of signaling pathways that promote tumor initiation and progression [18].

In cancer cells, including malignant plasma cells in multiple myeloma, ROS production is markedly increased as a consequence of altered metabolism, oncogenic signaling, and stress responses. Several intracellular compartments contribute to the elevated ROS burden. Mitochondria represent the predominant source, accounting for approximately 80% of intracellular ROS. During oxidative phosphorylation, incomplete reduction of oxygen in the electron transport chain generates superoxide anions, a process exacerbated by mitochondrial dysfunction frequently observed in cancer cells [19].

Another major source of ROS is the endoplasmic reticulum (ER), where oxidative protein folding and disulfide bond formation are mediated by oxidoreductases such as endoplasmic reticulum oxidoreductin 1 (ERO1) and protein disulfide isomerase (PDI). These reactions generate substantial amounts of hydrogen peroxide. In secretory malignancies such as multiple myeloma, characterized by excessive immunoglobulin production, ER-derived ROS play a particularly prominent role [10,20]. Additional sources of ROS include NADPH oxidases (NOX), membrane-associated enzymes that deliberately generate ROS to support proliferative and proangiogenic signaling and are often upregulated in cancer cells [10,21]. Peroxisomes also contribute through hydrogen peroxide production during fatty acid β-oxidation [22]. Furthermore, enzymes such as xanthine oxidase, cyclooxygenases, lipoxygenases, and nitric oxide synthases (NOS) expand the cellular pool of reactive oxygen and nitrogen species [23].

To counterbalance excessive ROS accumulation, cells are equipped with a complex antioxidant defense system comprising enzymatic and non-enzymatic components. Enzymatic antioxidants include superoxide dismutases (SOD), which catalyze the conversion of superoxide anions into hydrogen peroxide; catalase, which decomposes hydrogen peroxide into water and oxygen, primarily within peroxisomes; glutathione peroxidases (GPX), which reduce hydrogen peroxide and lipid peroxides using glutathione as a cofactor; and the thioredoxin (Trx/TrxR) system, which participates in peroxide detoxification and redox regulation of proteins. Among these enzymes, peroxiredoxins (PRDX) play a particularly important role. PRDX6 is unique within this family due to its dual peroxidase and phospholipase activities and has emerged as a key regulator of redox homeostasis in multiple myeloma cells [24,25].

Non-enzymatic antioxidants further support redox balance and include glutathione (GSH), the principal intracellular redox buffer, as well as exogenous antioxidants such as vitamins C and E. Endogenous low-molecular-weight compounds, including uric acid, bilirubin, and melatonin, also contribute to scavenging reactive species and limiting oxidative damage [24,26].

The dynamic equilibrium between ROS generation and antioxidant capacity defines the cellular “oxidative threshold.” ROS function as second messengers in multiple signaling pathways, including mitogen-activated protein kinase (MAPK), phosphatidylinositol-3-kinase/ protein kinase B (PI3K/AKT), and nuclear factor kappa-light-chain-enhancer of activated B cells (NF-κB), thereby influencing cell fate decisions. However, when ROS levels exceed the buffering capacity of antioxidant systems, irreversible oxidative damage occurs, triggering cell death pathways such as apoptosis or ferroptosis [25,26].

In cancer cells, redox homeostasis is characteristically shifted toward a state of persistently elevated ROS. Increased metabolic activity, mitochondrial dysfunction, and ER stress result in chronic oxidative pressure, while antioxidant systems are simultaneously upregulated to prevent ROS levels from surpassing the toxic threshold. This delicate balance allows cancer cells to exploit ROS-mediated signaling while avoiding oxidative catastrophe. Importantly, this state of “redox adaptation” creates a therapeutic vulnerability, as further ROS induction or disruption of antioxidant defenses can push cancer cells beyond their tolerance limit and selectively induce cell death [16,18].

## 3. Oxidative Stress in the Pathogenesis of Multiple Myeloma

The pathogenesis of multiple myeloma is a multistage process, encompassing the transformation of clonal plasma cells, their gradual accumulation in the bone marrow, and the development of complex interactions with the microenvironment. At the heart of this process is a disturbed redox balance. Plasma cells are already among the most metabolically active cells in the body, responsible for the massive production of immunoglobulins [1,9,10]. Under conditions of malignant transformation, the intensity of protein synthesis is further increased, leading to chronic endoplasmic reticulum stress. The folding and maturation of immunoglobulins in the endoplasmic reticulum requires numerous oxidation-reduction reactions mediated by oxidoreductases such as ERO1 and PDI [27]. These reactions are accompanied by the generation of hydrogen peroxide, and its accumulation contributes to elevated intracellular levels of reactive oxygen species. This oxidative stress, in turn, triggers the unfolded protein response (UPR) signaling cascade [27,28].

The UPR is adaptive in plasma cells and, under physiological conditions, allows for increased secretory capacity. However, in myeloma cells, this mechanism is reprogrammed toward chronic stress tolerance. The early phase of UPR activation induces the expression of protein chaperones and detoxification enzymes, which reduce ROS toxicity. However, when this response becomes chronic, permanent changes in cell signaling occur. Pathways that promote proliferation and resistance to apoptosis, such as NF-κB and MAPK, are activated, and increased ROS stabilize transcription factors, including Hypoxia-inducible factor 1-alpha (HIF-1α), further enhancing the metabolic adaptation of cancer cells to hypoxia [27,29].

Hypoxia, typical of the bone marrow microenvironment, is another important factor generating oxidative stress. Paradoxically, oxygen deficiency does not limit free radical production but increases it through dysfunction of the mitochondrial respiratory chain. Stabilization of HIF-1α also induces metabolic reprogramming toward aerobic glycolysis and activates genes encoding antioxidant proteins, such as HMOX1. As a result, myeloma cells gain the ability to adapt to a ROS-rich environment while simultaneously utilizing oxidative signals to promote angiogenesis, migration, and bone marrow interactions [30,31].

The impact of oxidative stress on genomic instability is also a key element in the pathogenesis of MM. ROS cause numerous DNA damages, including double-strand breaks and oxidative modifications of nitrogenous bases. Deregulation of DNA repair mechanisms is often observed in myeloma cells, leading to the accumulation of mutations more rapidly than in normal cells. This creates a vicious cycle in which oxidative stress promotes genomic instability, which in turn facilitates the development of treatment-resistant and clinically more aggressive subclones [32,33,34].

Interactions between ROS and the bone marrow microenvironment are equally important [35]. High levels of free radicals produced by myeloma cells affect neighboring stromal cells, osteoblasts, and osteoclasts, leading to impaired bone homeostasis. ROS activate osteoclastogenesis and promote bone resorption while simultaneously inhibiting osteoblast differentiation. This results in the formation of osteolytic lesions characteristic of multiple myeloma. Furthermore, oxidative stress in the bone marrow microenvironment alters the functioning of immune cells. T lymphocytes and Natural Killer (NK) cells lose some of their cytotoxic activity in conditions of excess ROS, which facilitates MM cell evasion of immune surveillance [1,10].

From the perspective of oxidative stress, the pathogenesis of MM thus appears to be a complex network of interdependencies. On the one hand, chronic ROS production is an inevitable consequence of immunoglobulin hypersecretion and cancer cell metabolism. On the other hand, ROS, by activating adaptive responses, enable these cells to survive in conditions that would normally lead to apoptosis. This creates a paradox: oxidative stress, potentially toxic and proapoptotic, is transformed by myeloma cells into a factor supporting their survival, progression, and clinical aggressiveness.

## 4. Adaptation Mechanisms and Drug Resistance

One of the most intriguing aspects of multiple myeloma biology is the ability of cancer cells to function in an environment of chronic oxidative stress and utilize it as a factor supporting survival. Crucial here is the activation of a series of adaptive mechanisms that, on the one hand, maintain reactive oxygen species levels at the toxic limit and, on the other hand, activate signaling pathways promoting proliferation and treatment resistance [1].

A central element of this response is the NRF2 (nuclear factor erythroid 2-related factor 2) pathway. Under basal conditions, NRF2 remains bound to the Keap1 protein and is degraded in the proteasome. However, under the influence of ROS, cysteines in Kelch-like ECH-associated protein 1 (Keap1) are modified, releasing NRF2 and enabling its translocation to the nucleus. There, it activates the expression of numerous genes encoding antioxidant enzymes, including SOD, catalase, glutathione peroxidase, and glutathione biosynthetic enzymes [36]. In myeloma cells, activation of NRF2 not only increases the ability to neutralize ROS but also promotes cellular metabolism and resistance to ER stress [37,38]. Clinical data indicate that overexpression of NRF2-dependent genes correlates with poorer prognosis and shorter progression-free survival, making this pathway a key determinant of redox adaptation in MM [39,40].

Another important mechanism is the activation of HMOX1 (heme oxygenase-1), an enzyme induced by both hypoxia and ROS. HMOX1 degrades heme into biliverdin, free iron, and carbon monoxide, each of which exerts significant biological effects [41]. In myeloma cells, HMOX1 exerts a protective effect by reducing excess ROS and increasing tolerance to proteasome inhibitors, including bortezomib [42]. It has been shown that, under hypoxic conditions, increased HMOX1 expression promotes cancer cell survival, while its suppression increases sensitivity to cytotoxic drugs. This indicates that the hypoxia-ROS-HMOX1 axis is one of the key adaptive mechanisms of myeloma cells in the bone marrow microenvironment [30].

Peroxidoxin 6 (PRDX6), an enzyme with unique properties combining peroxidase and phospholipase activity, has attracted particular attention in recent years. Under physiological conditions, PRDX6 protects against oxidative damage to membrane lipids and stabilizes mitochondrial function. Its overexpression has been demonstrated in multiple myeloma, which correlates with clinical progression and poor prognosis. PRDX6 knockdown in cell models leads to ROS accumulation, activation of c-Jun N-terminal kinase (JNK) and p38 kinases, decreased mitochondrial membrane potential, and inhibition of oxidative phosphorylation, and, consequently, cell apoptosis [43]. Recent studies have shown that PRDX6 plays a key role not only in neutralizing ROS but also in inhibiting ferroptosis—a specific, iron-dependent type of cell death characterized by lipid peroxidation. In cell models of multiple myeloma, PRDX6 overexpression led to reduced levels of iron (Fe^2+^) and lipid peroxides, which protected cells from ferroptotic membrane damage. Conversely, PRDX6 silencing resulted in ROS and iron accumulation, membrane destabilization, activation of ferroptosis, and increased sensitivity to bortezomib. This mechanism also involves the interaction of PRDX6 with GPX4, a key enzymatic gatekeeper against lipid peroxidation [44]. Notably, PRDX6 overexpression may identify a subset of MM patients with enhanced redox buffering capacity and reduced sensitivity to proteasome inhibitors.

MicroRNAs play an equally important role in regulating the oxidative response. In recent years, numerous microRNA molecules with altered expression in MM have been described, modulating both antioxidant and proapoptotic mechanisms. Of particular importance is miR-224, regulated by the transcriptional co-activator with a PDZ-binding motif (TAZ), a co-activator of the Hippo pathway that inhibits cancer cell growth. Overexpression of TAZ leads to increased levels of miR-224, which in turn inhibits the NRF2 program, increasing MM cell sensitivity to oxidative stress. On the other hand, low TAZ levels are associated with a redox-resistant phenotype, and its restoration restores drug sensitivity. These observations suggest that microRNAs may be both markers of biological heterogeneity in MM and potential therapeutic targets [45,46,47].

Adaptation to oxidative stress also involves profound metabolic changes. Myeloma cells undergo reprogramming toward increased aerobic glycolysis and increased flow through the pentose phosphate pathway, which allows for the regeneration of NADPH—the main cofactor of antioxidant reactions. Additionally, mitochondrial remodeling occurs in cancer cells, promoting ROS tolerance and reducing sensitivity to oxidative damage [48].

In summary, adaptive mechanisms in multiple myeloma form a complex network involving transcription factors, enzymes, and other proteins. Antioxidants, microRNAs, and metabolic changes: Thanks to these, cancer cells can transform an environment of high oxidative stress into a condition favorable to their survival and progression. At the same time, these mechanisms are key determinants of drug resistance, as they enable the neutralization of ROS induced by proteasome inhibitors and other therapies. From a clinical perspective, these adaptive redox mechanisms help explain the emergence of resistance to proteasome inhibitors and immunomodulatory agents observed in relapsed or refractory multiple myeloma. Assessment of redox-related pathways, including NRF2- and PRDX6-dependent signaling, may therefore aid in identifying patients at higher risk of treatment failure. Understanding this adaptive change is therefore not only fundamental to a better understanding of MM biology but also a starting point for the development of new therapeutic strategies aimed at achieving redox balance.

## 5. Oxidative Stress Biomarkers

In recent years, increasing attention has been paid to identifying markers of oxidative stress, which could be useful in both the diagnosis and monitoring of multiple myeloma. There is growing evidence that the degree of redox imbalance correlates with disease progression, the risk of organ damage, and treatment response. Therefore, oxidative biomarkers can be an important complement to classic prognostic tools such as the International Staging System (ISS) or the Revised International Staging System (R-ISS) [9].

The most frequently studied are classic pro- and antioxidant parameters, which can be measured in biological material such as blood serum. Lipid peroxidation markers include malondialdehyde (MDA), whose elevated levels have been repeatedly demonstrated in patients with multiple myeloma. Similar importance is attributed to the measurement of nitric oxide (NO), an excess of which promotes the formation of reactive nitrogen derivatives, which exacerbate DNA and protein damage. In turn, the activity of antioxidant enzymes such as superoxide dismutase (SOD), catalase (CAT), and glutathione peroxidase (GSH-Px) is often reduced in MM patients compared to healthy individuals, reflecting depletion of antioxidant reserves. Multivariate analyses indicate that high levels of MDA and NO combined with low activity of SOD, GSH-Px, and catalase may independently differentiate MM patients from healthy individuals and constitute useful diagnostic indicators [1].

In addition to these classic parameters, new markers of oxidative damage are also being studied, which may provide more precise information about the course of the disease. These include advanced oxidative protein products, which are products of albumin modification in the presence of ROS, and advanced glycation end products (AGEs), the formation of which is increased under conditions of oxidative stress and chronic inflammation. Higher concentrations of AOPPs and AGEs have been found in patients with MM compared to those with Monoclonal gammopathy of undetermined significance (MGUS) and healthy controls, and their levels correlate with the presence of osteolytic lesions [49]. S-nitrosylated proteins, in turn, show significant differences between patients and healthy controls, suggesting that they may reflect the stage of the disease process [50]. Incorporating these markers into a diagnostic panel could enable not only disease detection but also early diagnosis of its complications.

An interesting proposal integrating various parameters is the so-called Oxidative Stress Score (OSS). This composite index, constructed based on several easily measurable biochemical parameters, has recently been proposed as a prognostic tool in patients with newly diagnosed MM [9]. Studies involving large cohorts of patients have shown that high OSS is associated with shorter overall survival and progression-free survival, and its prognostic value is comparable to the R-ISS and even superior to the classic Durie–Salmon system. Furthermore, patients with high OSS did not significantly benefit from combination therapy based on proteasome inhibitors and immunomodulatory agents compared to monotherapy, suggesting that the level of oxidative stress may determine sensitivity to standard treatment [9]. The summary of oxidative stress biomarkers is presented in Table 1.

From a clinical perspective, the possibility of using oxidative stress biomarkers to monitor therapy efficacy seems particularly attractive. Because many drugs used in MM, such as proteasome inhibitors and some natural products, act by increasing oxidative stress, determining redox biomarkers during treatment could help assess the degree of ROS induction and predict treatment response. Simultaneously, monitoring antioxidant markers could warn of potential toxicity to healthy cells [51].

In summary, biomarkers of oxidative stress in multiple myeloma include both classic indicators of lipid peroxidation and antioxidant enzyme activity, as well as novel oxidative protein modification products and composite tools such as OSS. Although most studies are still exploratory in nature, the collected data indicate that these markers may be a valuable complement to classic prognostic factors and may find application in personalizing MM therapy.

## 6. Therapeutic Strategies Based on the Modulation of ROS and Antioxidant Systems in MM

The knowledge that myeloma cells function under chronic oxidative stress and rely on extensive defense mechanisms opens up new therapeutic possibilities. Redox imbalances can be exploited as the “Achilles’ heel” of myeloma cells—excessive oxidative stress can lead to irreversible damage and apoptosis, while blocking these defense mechanisms deprives cancer cells of their ability to adapt. In recent years, several strategies have been developed that rely on modulating ROS levels and targeted manipulation of antioxidant systems. The first and best-known approach is the use of proteasome inhibitors, such as bortezomib, carfilzomib, or ixazomib [27,52,53,54,55]. These drugs block protein degradation in the cell, leading to the accumulation of misfolded immunoglobulin chains and a strong activation of the ER stress response [10]. This is accompanied by intense ROS production, which becomes a significant component of the cytotoxic effects of proteasome inhibitors. An increase in ROS has been shown to correlate with the efficacy of bortezomib, and reducing oxidative stress through pharmacological or genetic enhancement of antioxidant mechanisms reduces the sensitivity of myeloma cells to treatment [16,56]. This is one of the main reasons why redox adaptation underlies drug resistance to this class of drugs.

In parallel, strategies targeting antioxidant buffers are gaining increasing interest. Among these, peroxidoxin 6 (PRDX6) is particularly important [43]. Studies have shown that myeloma cells are characterized by its overexpression, and that silencing PRDX6 causes ROS accumulation, mitochondrial dysfunction, and apoptosis [57]. Furthermore, the absence of PRDX6 significantly enhances the action of bortezomib, suggesting therapeutic potential in combined proteasome and peroxidase blockade [56]. Therefore, PRDX6 appears not only as a prognostic biomarker but also as a potential molecular target in multiple myeloma therapy. An interesting complement is the use of natural pro-oxidant compounds [58]. An example is caffeic acid phenethyl ester (CAPE), a component of propolis, which induces MM cell apoptosis by depleting the glutathione pool and activating stress response genes such as HMOX1 and Growth Arrest and DNA Damage 45 (GADD45) [59,60]. The action of CAPE is based on a mechanical shift in the redox balance toward oxidative stress, leading to irreversible damage and cell death. It is worth emphasizing that the cytotoxic effect of CAPE can be reversed by supplementing glutathione and enhanced by blocking its synthesis, which clearly indicates the role of the antioxidant pool in determining cell fate [61,62]. Another natural compound is xanthohumol, a prenylated flavonoid derived from *Humulus lupulus* L. It inhibits cell proliferation and induces apoptosis in myeloma cells by producing reactive oxygen species, activating the JNK and Extracellular Signal-Regulated Kinases (ERRK) pathways, and inhibiting Vascular Endothelial Growth Factor (VEGF) production [58,63,64].

Similar properties are exhibited by an indole alkaloid extracted from the dried, unripe fruit of *Evodia rutaecarpa*, known as evodiamine (evo). It has been shown to activate caspase 3 and 9 in myeloma cells, increase cytochrome C expression, and contribute to the production of reactive oxygen species. This results in inhibition of myeloma cell proliferation and increased apoptosis [58,65,66]. Natural substances with redox-modulating effects may therefore act as adjuvants supporting standard treatment. Despite promising preclinical data, the clinical applicability of natural pro-oxidant compounds remains limited by issues related to bioavailability, dosing, and the lack of large prospective clinical trials.

**Figure 1:**
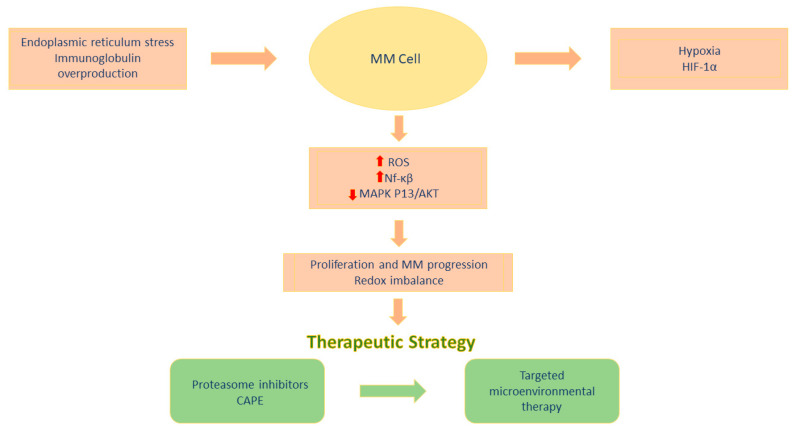
Oxidative stress and therapeutic strategies in multiple myeloma.

Another possibility is a combined therapeutic approach, which involves combining pro-oxidant drugs with conventional MM treatment regimens. This concept is based on the assumption that different classes of drugs can synergistically increase oxidative stress, exceeding the cancer cell’s tolerance threshold. An example is the observation that PRDX6 blockade enhances the cytotoxicity of bortezomib [56], and similar synergisms may occur when combining proteasome inhibitors with other pro-oxidants, such as evodiamine or inhibitors of antioxidant systems. This approach seems particularly promising for patients resistant to standard treatment, in whom redox adaptive mechanisms play a key role in maintaining cancer cell survival [44]. It is worth noting that modulation of oxidative stress carries a certain risk of toxicity. ROS, although lethal to cancer cells, can also damage healthy tissues, especially those with high metabolic activity, such as the heart or kidneys [67]. The effects of redox imbalance on MM cells and potential therapeutic strategies are shown in Figure 1. Therefore, future strategies should be based on a precise balance of therapeutic effect and safety, as well as the use of biomarkers that will allow monitoring the level of oxidative stress and tailor the intensity of treatment to the individual patient’s needs.

In summary, manipulating the redox balance in multiple myeloma represents an attractive therapeutic approach. Using ROS as effectors of cancer cell death, blocking antioxidant enzymes such as PRDX6, and incorporating natural pro-oxidants opens the prospects for personalized therapy based on the vulnerability of myeloma cells to oxidative stress.

## 7. Supportive Interventions

To date, research on oxidative stress in multiple myeloma has focused primarily on the role of drugs targeting redox balance, but increasing attention is also being paid to supportive interventions that can modulate ROS levels in the patient’s body. These primarily include physical activity, diet, and antioxidant supplementation. Although this topic remains under investigation, available data suggest that appropriately selected non-pharmacological strategies can improve patients’ quality of life and potentially influence the course of the disease [68,69].

Physical activity, long recommended for cancer patients as a form of rehabilitation, has also been evaluated for its effect on oxidative parameters in multiple myeloma. A randomized clinical trial demonstrated that a six-week Nordic walking training cycle in patients in myeloma remission led to reduced markers of oxidative damage and beneficial changes in iron metabolism. A regulation of gene expression related to iron homeostasis and a decrease in ferritin concentration were also observed, which may indicate reduced inflammation and oxidative stress. Importantly, the intervention was well tolerated and its safety was confirmed in older adults, who constitute the majority of patients with MM [70]. Aerobic exercise—such as brisk walking, swimming, or cycling—plays a particularly important role because it leads to a moderate, controlled increase in ROS production, which in turn stimulates endogenous antioxidant mechanisms [71]. This phenomenon is referred to as oxidative hormesis—slight, repeated oxidative stress induced by physical exercise stimulates the expression of defensive enzymes such as superoxide dismutase (SOD) and glutathione peroxidase (GPX), increasing overall cellular resistance to oxidative damage. Additionally, aerobic activity improves tissue oxygenation, increases mitochondrial efficiency, and regulates glutathione metabolism, which helps stabilize redox balance [72].

From the perspective of MM patients, it is also important that aerobic training can influence the bone marrow microenvironment. Improved circulation and oxygenation promote hematopoietic renewal, while reduced oxidative stress and inflammation limit the activation of osteoclasts responsible for bone destruction. Regular moderate-intensity exercise can therefore counteract progressive sarcopenia, improve bone mineral density, and increase tolerance to cancer therapy. As a result, physical activity serves a dual function—it supports the rehabilitation process and simultaneously acts as a natural modulator of oxidative balance [73,74,75]. These results suggest that moderate physical activity, especially aerobic activity, may play not only a rehabilitative but also a biologically protective role, contributing to the reduction in oxidative stress and supporting multiple myeloma therapy. Maintaining physical activity during and after treatment supports the healing process, allows for greater fitness, and improves mental well-being.

Diet and nutrients are also a potential tool for regulating oxidative stress, and patients with multiple myeloma should follow general recommendations for a healthy diet. A proper diet may contribute to mitigating the side effects of treatment [69]. The literature emphasizes the role of foods rich in polyphenols (curcumin, resveratrol), antioxidant vitamins (C and E), and trace elements such as selenium and zinc, which participate in the functioning of antioxidant enzymes [1,76]. Polyphenols contained in dark berries reduce the production of ROS in cells, limit and interrupt free radical reactions, prevent DNA damage, and increase the activity of antioxidant enzymes. Consuming polyphenols increases the body’s enzymatic and non-enzymatic antioxidant barrier [77,78].

Curcumin has a wide range of therapeutic effects. It is known for its anticancer, antioxidant, anti-inflammatory, proapoptotic, and antiprogressive properties associated with metastasis and angiogenesis. Its antioxidant activity is comparable to that of vitamins C and E; it neutralizes reactive oxygen species (ROS) in cells, including the hydroxyl radical, nitric oxide, and superoxide anion radical, and also increases glutathione levels. This anti-inflammatory effect is due to, among other things: inhibiting NF-κB activation and reducing the expression of pro-inflammatory proteins (e.g., Interleukin 6 (IL-6), cyclooxygenase 2 (COX-2)). The anti-cancer activity of curcumin includes inhibiting tumor initiation and proliferation and decreasing the expression of pro-angiogenic proteins (VEGF, bFGF), which makes it a direct inhibitor of angiogenesis [79,80]. In myeloma cells, curcumin additionally targets key survival pathways: it inhibits constitutive and IL-6-induced Signal Transducer and Activator of Transcription 3 (STAT3) phosphorylation, and also quenches NF-κB (stabilization of Inhibitor of nuclear factor kappa B alpha (IκBα), inhibition of IκB kinase (IKK)), PI3K/Akt/mTOR, and MAPK/ERK; this results in a decrease in cyclin D1, MYC, and antiapoptotic proteins (B cell lymphoma 2 (Bcl-2), B cell lymphoma -extra large (Bcl-xl), myeloid cell leukemia 1 (MCL-1)), cycle arrest (G1/S or G0/G1), and activation of caspases (including 7 and 9). Importantly, in MM, curcumin may also act through the Notch3-p53 axis (↓ Notch3, ↑ p53/p21), and in the context of the microenvironment, reduce IL-6 secretion and limit angiogenesis by modulating VEGF-VEGFR2 signaling [81,82].

In combination therapies, synergism has been demonstrated between curcumin and proteasome inhibitors: bortezomib (including by inhibiting NF-κB and sensitizing the JNK pathway) and carfilzomib (stronger NF-κB silencing, increasing p53/p21, and arresting in G0/G1) [83,84].

The anticancer effect of resveratrol is based on its ability to block all three stages of the carcinogenesis process [85]. By scavenging free radicals, it inhibits the initiation of carcinogenesis. Furthermore, resveratrol is involved in the induction of quinone reductase, which in turn enables the detoxification of carcinogenic factors. During cancer progression, it reduces the activity of compounds belonging to the cytochrome 450 family and inhibits their expression. In the third phase of carcinogenesis, it exhibits cytotoxic effects by inhibiting DNA polymerase and ribonucleotide reductase. Resveratrol halts the cell cycle in the G1/S phase, resulting in apoptosis and thus inhibiting cancer cell proliferation. It is also a compound with strong antioxidant properties and the ability to scavenge free radicals [86,87]. In the context of multiple myeloma, resveratrol exerts cytotoxic effects by activating AMPK and simultaneously inhibiting the mTOR/p70S6K pathway, which leads to the initiation of autophagy and apoptosis in myeloma cells (U266, RPMI-8226, NCI-H929). It increases the expression of LC3-II and Beclin-1 and simultaneously decreases the level of the antiapoptotic protein Survivin, activating caspase-3 and leading to PARP degradation. Inhibition of autophagy partially abolishes this effect, indicating that resveratrol induces autophagy-associated apoptosis. Additionally, it inhibits NF-κB, increases p53 activity, and sensitizes cells to bortezomib and melphalan [88]. In clinical trials, the combination of resveratrol with copper (resveratrol–Cu) in patients undergoing autologous transplantation after high-dose melphalan significantly reduced the incidence of severe oral mucositis and lowered levels of the proinflammatory cytokines TNF-α and IL-1β, suggesting a tissue-protective effect and reduced inflammatory stress associated with therapy [89].

Another example is the triterpenoids found in apples and cranberries. These compounds contain oleanolic and ursolic acids. They block the production of inflammatory cytokines and are also potent activators of the Nrf2 pathway, thus potentially inhibiting nitric oxide synthase (NOS).

A potential compound with the ability to inhibit the proteasome and inhibit myeloma cell proliferation is gallic acid. Sources of this substance include bananas, strawberries, lemons, red wine, and green tea. It possesses strong antioxidant properties due to the presence of three hydroxyl groups attached to the aromatic ring in the ortho position [90,91,92,93].

Preclinical studies indicate that natural compounds, such as caffeic acid phenethyl ester (CAPE), may increase oxidative stress in cancer cells and act synergistically with proteasome inhibitors. Although these results are promising, there are no large clinical trials that clearly confirm the effectiveness of dietary therapy or supplementation in modulating the course of MM. Furthermore, caution is warranted, as excessive antioxidant supplementation could theoretically reduce the effect of ROS-generating drugs, such as bortezomib.

The role of interventions targeting the gut microbiota, which may indirectly influence oxidative balance by modulating inflammatory processes and metabolism, is also of interest. Preliminary observations indicate that a high-fiber diet (25–35 g per day) and probiotics may improve metabolic parameters and reduce markers of oxidative stress in cancer patients, although specific data for multiple myeloma remain very limited [69,94]. It has been suggested that the gut microbiota may play a role in the pathogenesis of multiple myeloma, specifically disturbances in its composition known as dysbiosis. This may influence the development of inflammation and metabolite production, thus impairing the functioning of the immune system. Depending on the body’s condition (age, diseases, nutritional status), the composition of the microbiota changes, inducing the development of diseases, affecting the regulation of the immune response or drug interactions. A high-carbohydrate, low-fiber diet with a high red meat content alters the gut microbiota and promotes the development of cancer. Unfavorable changes in gut flora may increase susceptibility to infections in hematological diseases or impair response to chemotherapy or immunotherapy [95]. Table 2 summarizes current evidence linking specific gut bacterial taxa with multiple myeloma progression, treatment response, and clinical outcomes. The reported associations derive from a combination of preclinical models, observational human cohorts, and recent metabolomics-integrated microbiome studies. Collectively, these data indicate that gut microbiota composition influences multiple myeloma biology through immune modulation, metabolic reprogramming, and regulation of inflammatory signaling pathways.

Several bacterial taxa have been associated with disease-promoting effects. In particular, *Prevotella heparinolytica* has been shown in murine models to induce Th17 cell differentiation and IL-17-dependent inflammatory signaling within the bone marrow microenvironment, thereby accelerating myeloma progression. Similarly, enrichment of nitrogen-recycling bacteria, including *Klebsiella pneumoniae* and *Streptococcus* spp., has been observed in patients with multiple myeloma and is linked to enhanced glutamine availability supporting malignant plasma cell metabolism [95,107]. In contrast, short-chain fatty acid-producing bacteria are generally associated with more favorable disease characteristics. Reduced abundance of butyrate-producing taxa, such as *Anaerostipes hadrus* and *Clostridium saccharobutylicum*, has been consistently reported in multiple myeloma, suggesting loss of SCFA-mediated anti-inflammatory and immunoregulatory signaling. Experimental supplementation with *Clostridium butyricum* has been shown to mitigate tumor progression in preclinical models, supporting a potential protective role for SCFA-producing bacteria [95,110]. Among clinically relevant taxa, *Faecalibacterium prausnitzii* emerges as a robust prognostic marker. Higher baseline abundance of this species has been independently associated with improved progression-free and overall survival following autologous hematopoietic stem cell transplantation, as well as reduced transplant-related gastrointestinal toxicity. These findings underscore the importance of gut microbial integrity in the peri-transplant setting [109]. The role of *Bacteroides* spp. appears to be context-dependent. While these bacteria contribute to gut barrier maintenance and immune tolerance through polysaccharide A-mediated regulatory T-cell induction, their immunomodulatory effects may also attenuate antitumor immune responses in certain oncologic contexts. Recent studies further demonstrate that *Bacteroides* abundance is modifiable through lifestyle-based interventions, including structured physical activity and time-restricted eating, highlighting their potential relevance for non-pharmacological microbiome modulation in multiple myeloma patients [111,112,117]. Importantly, recent metabolomics-integrated analyses refine the interpretation of *Ruminococcus* spp. Rather than serving solely as markers of dysbiosis, *Ruminococcus* spp. positively correlate with the production of microbiota-derived urolithins, particularly urolithin A, a metabolite with demonstrated antimyeloma activity in vitro, ex vivo, and in vivo. Reduced abundance of *Ruminococcus* has been observed in relapsed or refractory disease, suggesting a potential protective role that is dependent on disease stage and associated microbial metabolite profiles [115]. By producing SCFAs, the gut microbiota enhances antioxidant enzyme expression, suppresses ROS production, and improves mitochondrial function, thereby reducing oxidative stress and limiting multiple myeloma progression.

Overall, the data summarized in Table 2 shows that gut microbiota alterations in multiple myeloma are not always beneficial or detrimental but rather reflect a dynamic and context-dependent ecosystem, in which specific bacteria and their metabolites jointly influence immune regulation, tumor metabolism, treatment tolerance, and clinical outcomes. These findings support further investigation of microbiome- and metabolite-targeted strategies as adjunctive approaches in multiple myeloma management.

In summary, supportive interventions—including physical activity, dietary modifications, and targeted supplementation—appear to be promising adjuncts to multiple myeloma therapy, particularly in the context of modulating oxidative stress. Their potential benefits include not only reducing oxidative damage and improving quality of life but also potentially synergizing with pharmacological treatments. Despite promising preclinical data, robust clinical evidence supporting antioxidant supplementation in MM remains limited. Therefore, further research is needed to determine the optimal forms, doses, and duration of such interventions, as well as their impact on survival and disease progression.

## 8. Conclusions and Future Directions in Redox Targeting

Oxidative stress occupies a central place in the biology of multiple myeloma, determining both the development of the disease, its clinical course, and response to treatment. Plasma cells, due to their physiological function of massively producing immunoglobulins, are naturally exposed to high oxidative stress. Neoplastic transformation and cell accumulation in the hypoxic bone marrow microenvironment further exacerbate this condition, leading to the permanent activation of pathways related to the generation and neutralization of reactive oxygen species.

The paradox is that while excess ROS is potentially toxic and could lead to apoptosis, in multiple myeloma, it has been transformed into an adaptive mechanism. Activation of the NRF2 and HMOX1 pathways, overexpression of enzymes such as PRDX6, and metabolic remodeling toward intensive glycolysis and the pentose phosphate pathway allow myeloma cells to function at the edge of redox tolerance. Moreover, oxidative stress serves a signaling function—stabilizing HIF-1α and activating NF-κB, MAPK, and other pathways that support proliferation, angiogenesis, osteoclastogenesis, and immunosuppression in the bone marrow microenvironment. As a result, ROS become a factor contributing to cancer progression and the development of complications such as bone damage and renal failure.

Equally promising are therapeutic strategies based on modulating the redox balance. Proteasome inhibitors already utilize oxidative stress as part of their mechanism of action, and their effectiveness can potentially be enhanced by blocking antioxidant buffers such as PRDX6. Natural compounds, including CAPE, may act synergistically with standard therapy, shifting the balance toward the pro-oxidant side and inducing cancer cell apoptosis. At the same time, there is growing awareness that excessive suppression of ROS, for example, through uncontrolled antioxidant supplementation, could impair the efficacy of pro-oxidant-based treatments, emphasizing the need for a balanced approach.

Several key challenges loom on the research horizon. First, validation of PRDX6 as a therapeutic target and biomarker is essential—the results of preclinical studies are very promising but require translation to animal models and, in the long term, clinical trials. Secondly, it is necessary to develop combination strategies that will utilize oxidative stress to eliminate cancer cells while limiting toxicity to healthy tissues. Thirdly, it is worth considering the potential of non-pharmacological interventions, such as physical activity and diet, which can support redox balance and improve patients’ quality of life. Fourthly, the importance of individual genetic and epigenetic differences in shaping oxidative response and treatment sensitivity remains an open question—better understanding these differences could enable personalized therapy based on a specific patient’s redox profile.

Future research should focus on translating redox biology into clinically applicable strategies in multiple myeloma. In particular, the identification and validation of disease-specific oxidative stress biomarkers, such as PRDX6-related signatures or composite indices like the Oxidative Stress Score, may improve risk stratification and therapeutic decision-making. Further studies are needed to explore combination approaches that exploit redox vulnerabilities, including the simultaneous induction of ROS and inhibition of antioxidant defenses. Emerging biomarkers of DNA and lipid damage (e.g., 8-OHdG), together with integrative “omics” approaches such as metabolomics and proteomics, offer a more comprehensive and system-level assessment of oxidative stress. In addition, a deeper understanding of the interplay between oxidative stress, metabolic reprogramming, and the bone marrow microenvironment—especially the role of the gut microbiota—may open new avenues for personalized and supportive therapies. Ultimately, integrating redox-based biomarkers with clinical parameters could contribute to the development of precision medicine strategies in MM.

In summary, oxidative stress in multiple myeloma is a dual phenomenon: it promotes disease development and progression but also represents a potential therapeutic target. It is this ambivalence that makes redox research in multiple myeloma exceptionally important, both from a cognitive and practical perspective. The future belongs to personalized therapies, in which oxidative stress biomarkers will be used to stratify patients and monitor treatment, and new drugs modulating redox balance will help overcome resistance and improve the prognosis in this still incurable disease. Targeting redox adaptation may represent one of the most rational strategies to overcome therapeutic resistance in multiple myeloma.

### Limitations

This review has several limitations that should be acknowledged. First, it is based predominantly on preclinical studies and observational clinical data, as robust prospective trials specifically addressing redox-targeted interventions in multiple myeloma remain limited. Second, the heterogeneity of methodologies used to assess oxidative stress biomarkers complicates direct comparison across studies and limits immediate clinical translation. Third, evidence linking gut microbiota composition with multiple myeloma progression and treatment response is still emerging and largely associative. Finally, the complex and context-dependent nature of redox biology implies that therapeutic modulation of oxidative stress may exert variable effects depending on disease stage, treatment background, and patient-specific factors. These limitations highlight the need for standardized biomarker validation and well-designed clinical studies to translate redox-based concepts into clinical practice.

## Figures and Tables

**Table 1:** Selected oxidative stress biomarkers in multiple myeloma and their clinical relevance.

Biomarker	Biological Material	Change in MM	Clinical Relevance	Ref.
MDA	Serum/plasma	increased	Marker of lipid peroxidation; correlates with disease activity and oxidative burden	[1,49]
NO	Serum	increased	Reflects nitrosative stress; associated with protein and DNA damage	[1]
AOPPs	Serum	increased	Associates with bone disease and inflammatory status	[49]
AGEs	Serum	increased	Correlates with oxidative stress and chronic inflammation	[49]
SOD	Serum/erythrocytes	decreased	Reflects depletion of enzymatic antioxidant defense	[1]
CAT	Serum/erythrocytes	decreased	Indicates impaired hydrogen peroxide detoxification	[1]
GPX	Serum/erythrocytes	decreased	Marker of reduced antioxidant capacity	[1]
OSS	Composite index (serum-based parameters)	increased	Predicts overall survival (OS) and progression-free survival (PFS); complements ISS and R-ISS	[9]

**Table 2:** Gut microbiota associated with multiple myeloma progression and clinical outcomes.

Bacterial Taxon	Association with Multiple Myeloma	Main Mechanisms	Evidence Type	Ref.
*Prevotella heparinolytica*	Promotes disease progression	Induction of Th17 cells and IL-17-driven inflammatory signaling in bone marrow	Preclinical (murine MM models)	[96,97]
*Klebsiella pneumoniae*	Associated with accelerated MM progression	Nitrogen recycling and de novo glutamine synthesis supporting plasma cell metabolism	Preclinical; patient-associated enrichment	[98,99,100]
*Streptococcus* spp.	Increased abundance in MM patients	Enhanced nitrogen utilization and glutamine availability, promoted proliferation of myeloma cells, induced chemotherapy resistance	Observational (human cohorts)	[99,101,102,103]
*Clostridium butyricum*	Potential protective effect	Butyrate production; suppression of NF-κB, pro-inflammatory cytokines and bone marrow inflammation	Preclinical (mouse model), Clinical	[104,105,106]
*Anaerostipes hadrus*	Reduced abundance in MM	Loss of short-chain fatty acid-mediated anti-inflammatory signaling	Observational	[99]
*Clostridium saccharobutylicum*	Reduced in MM patients	Decreased butyrate production	Observational	[99]
*Eubacterium hallii*	Associated with favorable treatment response	SCFA production; correlation with MRD negativity	Observational (post-induction therapy)	[106,107,108]
*Faecalibacterium prausnitzii*	Higher pre-transplant abundance is associated with improved progression-free and overall survival after autologous HSCT; lower abundance predicts inferior Progression free and overall survival	SCFA (butyrate) production; anti-inflammatory effects; modulation of gut–bone marrow immune axis and transplant-related toxicity	Observational (HSCT cohorts), Clinical	[95,109,110]
*Bacteroides* spp.	Abundance is modifiable by lifestyle interventions in MM patients; higher abundance associated with preserved gut barrier function and reduced gastrointestinal toxicity, but potentially inferior antitumor immune responses in some oncologic settings	Polysaccharide A-mediated Treg induction via TLR2 signaling; modulation of gut barrier integrity and immune tolerance	Observational clinical studies; lifestyle intervention study; mechanistic immunology	[111,112]
*Blautia* spp.	Associated with increased transplant-related toxicity	Dysbiosis-related inflammation	Observational (HSCT)	[97,113,114]
*Ruminococcus* spp.	Context-dependent association with MM; reduced abundance observed at relapse, while higher abundance correlates with urolithin production and improved progression-free survival	Contribution to polyphenol metabolism and urolithin A production; indirect modulation of tumor metabolism and immune responses	Observational clinical study; metabolomics-integrated microbiome analysis	[108,115,116]

## Data Availability

No new data were created or analyzed in this study. Data sharing is not applicable to this article.
